# A Case of Reproduction of the Inferior Maxillary Bone

**Published:** 1853-07

**Authors:** Wm. Jones

**Affiliations:** Kenton, Ohio.


ARTICLE V.

### *A Case of Reproduction of the Inferior Maxillary Bone.* By Wm. Jones, M. D., Kintre, Ohio.

Some years ago I was called to give some medical advice to Mrs. B., a lady of thirty-five, residing near Bellefontaine, Lagare county, Ohio. I found her suffering under most if not all the worst effects of mercury combined. On inquiry, I was informed that she had been severely salivated about five years previously, by a single dose of calomel, which she took from a physician for the purpose of correcting a bilious derangement. Not long after the drug was swallowed and before it had time to operate as a cathartic, she had the temerity to go to a fine cold spring of water from which she drank freely, at the same time indulging freely in its application to the head, face, upper and lower extremities, all of which resulted in a hastily developed mercurial action of great violence, accompanied by much pain and swelling of the face, delirium and other alarming constitutional disturbances which ran on for sometime threatening to destroy her life. The constitution finally survived the shock, the severity of the mercurial excitement having passed off, leaving her the subject of extreme exhaustion, with a profuse ptyalism and a necrosis of the inferior maxillary bone, which had acted as a drain upon her feeble remains of vitality up to the time of my visit, and for many weary years afterwards, as the sequel will show.

I should add, this misfortune befel her in some part of Indiana, where she was then living. She was a most pitiful object of suffering—the merest wreck of human existence—haggard, squalled and disgusting, not more in appearance than on account of the highly offensive smell constantly issuing from her mouth, polluting the entire atmosphere in her vicinity, and rendering her almost inapproachable.

On close examination I perceived there was a complete necrosis of the horizontal portion of the inferior maxillary bone, on both sides of the face, from a point as far back as the sapientiæ molares, involving the body of the bone from the angles forward. This portion of the jaw was in a state of nudity, wholly divested of the periosteum, of a dark color, cracked and fissured in almost every direction, and ready to fall to pieces upon the slightest touch. It was held together by the arch of the teeth still present, having a slight hold in the alveoli, and kept in their position by many turns of wire which had been ingeniously interwoven among them by some person for this purpose; the pressure of the cheeks and tongue afforded some lateral support to the crumbling mass. A little force applied under the chin would displace all by raising it up, and the same exerted by the application of the finger farther back under the jaw, on either side, gave the clearest evidence that the dead bone was already detached from the living portion, consisting of but little more than the ramus, with the condyles and coronoid processes. She assured me, that the finger could be passed under the bone within the mouth without meeting resistance from parts adhering at any point, and that the whole might be lifted out of the mouth together, without the necessity of an operation of any kind, which I thought might be the case.

So great was the constitutional deterioration, that her sight, hearing and other senses were much obtruded, and the intellectual faculties almost reduced to a blank. The disease was at this time passive, unattended with pain or other inconvenience than was inseparable from the continued flow of saliva which was excessive still, and the disgusting embarrassments consequent upon the necrosis. I directed an occasional draught of lime water, with the free use of tonic bitters and liberal diet, as the best means of promoting her general health and of enabling her to bear up under the loathsome burden until time should relieve her of it. It is a remarkable fact, that though it was apparent both to herself and me, that the rotten mass with its filthy odor might have been removed without pain or difficulty and replaced by some less offensive substitute, which I suggested to her at the time, she clung to it with the utmost tenacity, and dreaded the loss of it exceedingly, both on account of the little service it yet afforded her in speaking and the management of her food and the deformity which might be apprehended from its removal, without the certainty of a successful substitute. Her health was somewhat benefited and gradually improved, and time brought away the *reluctant jaw* with its appendages.

Some three or four years had elapsed when I saw her again, and at this time the dead bone and teeth were entirely gone, the face was kept in shape by means of a roll of linen or cotton rags of sufficient size and firmness to keep the chin at a suitable degree of forward projection, rising high enough to keep the lip from falling in, and the tongue from protruding out of the mouth. Thus she contrived to get on for some years longer, when she removed and came to the western part of this (Harden) county, where I at that time practiced medicine, which afforded me frequent opportunities of seeing her. Observing a very great change in her appearance for the better I endeavored to learn by special inquiry how far her loss had been repaired. I found she yet employed a small linen or cotton roll to aid in supporting the cheeks and lip, but the chin was fairly maintained in place at the natural degree of projection forward, by a NEW MAXILLARY BONE, or something acting as an equivalent. I thought it true bone, answering in firmness, strength and general conformation to the original jaw, appearing to have the same muscular attachments, and fulfilling its uses, so far as this could be done without the teeth. It approximated the natural one in shape and dimensions, after the teeth and alveolar process have been removed as in aged persons, but was roundish and not more than three-eighths of an inch in thickness, about midway between the angles and chin. It was duly invested with the proper coverings. Not a vestige of the old bone or lower set of teeth was visible, though I believe she retained most of the upper set in tolerable preservation, the mischief having principally fallen on the lower jaw. The flow of saliva was but little in excess, and her health and spirits quite renovated. She thought that the new jaw was a reproduction of the bone, of which there was to appearance much satisfactory evidence in the developments of the case. She thought the new formation commenced at the chin, and the renovating extremities of the old jaw, and progressively but slowly met in the centre upon both sides, where the bone was most slender, and acquired less of the form of the original. This would seem the more plausible, when we remember the totally necrosed and exfoliated condition of the bone, being fairly separated from the living fragment on either side, and destitute of all vitality for years, before the new bone appeared and even dismissed from the mouth and replaced by the rag roll, which was long found necessary to keep the chin projected forward, and give any tolerable degree of shape to the face, before it was apprehended that the new bone would be produced. I regret I had not more frequent opportunities of observing the progress of the case. From all I was able to gather in reference to it, I thought, and continue to regard it, a case of genuine reproduction of the inferior maxillary bone so far as effected.

I am aware that the reproduction of the maxillary bones in whole or in part is held to be doubtful, if at all possible, and, that there is not a well authenticated case of the kind on record. This is the first and only case with which I have had an acquaintance, I cannot class it among the cases alluded to by Stanley, in which he speaks of a "gray pumice-like osseous substance, deposited round the necrosed bone, from an inflamed periosteum, and made up wholly of disorganized matter." In this case the new bone was not produced while the periosteum was in a state of inflammation, nor until the patient was measurably restored to health, and the system generally had undergone considerable renovation. It does not appear to have been the spurious offspring of a hot and hasty freak of nature, but the legitimate produce of a *cool, deliberate, well intentioned* resolve and purpose, pertinaciously adhered to until accomplished, though by no means begun in a hurry, as the history of the case abundantly shows; and it was cleverly and handsomely done, conferring on the sufferer an instrument of great usefulness, whilst it did much to remedy her deformity, and make her life not only tolerable, but desirable. It doubtless differed much from the original in its organic structure, (as what new production of bone does not,) but was nevertheless essentially bone, and not a mere calculous or concrete substance. It was endowed with the elements of vitality, and possessed vital and functional relations to the neighboring parts, which were happily maintained through the remainder of life, this could not have been the case if it had been a mere calcareous deposit, a concrete or foreign substance. The lady lived in the enjoyment of a good degree of health and cheerfulness until recently. I believe she is now dead, having died of some other disease.

